# Diagnostic Value of PET/MR with DWI for Burkitt Lymphoma

**DOI:** 10.3390/diagnostics11101867

**Published:** 2021-10-11

**Authors:** Chiara Giraudo, Rossella Simeone, Margherita Fosio, Dario Marino, Diego Cecchin

**Affiliations:** 1 Department of Medicine—DIMED, University of Padova, 35122 Padova, Italy; margherita.fosio@gmail.com; 2 Nuclear Medicine Unit, Department of Medicine—DIMED, University of Padova, 35122 Padova, Italy; rossella.simeone@aopd.veneto.it (R.S.); diego.cecchin@unipd.it (D.C.); 3 Medical Oncology I, Veneto Institute of Oncology IOV—IRCSS, 35122 Padova, Italy; dario.marino@iov.veneto.it

**Keywords:** Burkitt lymphoma, PET/MR, adults, staging

## Abstract

18F-FDG-PET/MR images, including DWI, of a 46-year-old male admitted to the Emergency Room of our tertiary center, who was suffering from diplopia, left orbital pain, and a headache for two weeks, demonstrated multiple hepatic nodules, a pancreatic mass, and skeletal metastases, in addition to thrombosis of the left cavernous sinus, thickening of the small intestine, and a large hepatic lesion identified at head and neck MR and whole-body CT, respectively. Hepatic and bone marrow biopsies revealed the diagnosis of Burkitt lymphoma. After four cycles of rituximab, cyclophosphamide, doxorubicin, vincristine, methotrexate/ifosfamide, etoposide, and high dose cytarabine (R- CODOX-M/IVAC), a complete metabolic response occurred.

Burkitt lymphoma is a very aggressive B-cell lymphoma rarely affecting adults (i.e., <1% of non-Hodgkin lymphomas) and mainly involving the abdomen [1,2,3]. In particular, the bowel is mostly affected whereas the liver is usually a secondary site, and the pancreas is rarely involved. Burkitt lymphoma of the bone marrow occurs in around 30% of the cases and is usually associated with poor prognosis. Whole-body techniques, such as computed tomography (CT) and 18F-fluorodeoxyglucose (FDG)- positron emission tomography (PET)/CT are crucial in the diagnostic process [2,4,5]. In the latter in particular, providing metabolic information is very useful for detecting both nodal and extra-nodal lesions, predicting the outcome, and evaluating the response to treatment. 

Although PET/magnetic resonance (MR), including diffusion weighted imaging (DWI), demonstrated to be very accurate for lymphomas in general, its current application for Burkitt lymphoma is scarce [2,6,7,8]. In our case, PET/MR demonstrated to be very useful since functional and metabolic information, simultaneously collected, revealed multiple lesions (i.e., hepatic, pancreatic, and skeletal) that were not visible otherwise, on CT or basic MR sequences (Figure 1). Moreover, it should be highlighted that our patient represents a rare case of full response to treatment since, typically, the survival rate in adults is usually low (i.e., 33% of 5-year survival rate in patients older than 60) (Figure 1) [9,10].

## Figures and Tables

**Figure 1:**
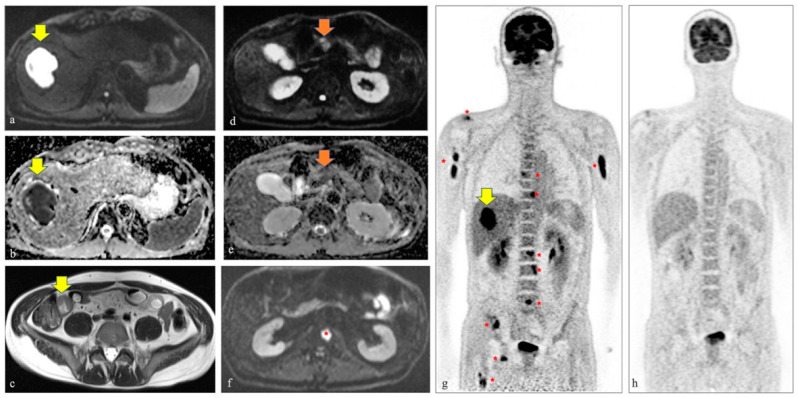
A 46-year-old male was admitted to the Emergency Room of our tertiary center because of diplopia, left orbital pain, and a two-week headache. At clinical assessment, ptosis and left III and VI cranial nerve palsy were diagnosed. The laboratory tests were unremarkable, except for high levels of lactate dehydrogenase. An MR scan of the head and neck demonstrated thrombosis of the left cavernous sinus. Then, the patient underwent a whole-body contrast enhanced CT and a thickening of the small intestine and a large hepatic lesion, although not typical for any specific disease, were identified. Given the not univocal findings at CT and aiming to better characterize the hepatic and intestinal lesions, also in terms of metabolic activity, the referring clinicians decided to move further with the diagnostic workflow. A few days later, a 18F-FDG-PET/MR including DWI was performed and confirmed the above-mentioned lesions. In particular, on the axial DWI image (i.e., b value 800) and corresponding ADC map in (**a**,**b**) the restricted diffusion of the liver lesion is well seen (yellow arrows), while in (**g**) the high metabolic activity at PET of the same hepatic lesion is detected (yellow arrow on the coronal map of the PET component). Moreover, on the axial T2 half-Fourier single-shot turbo spin-echo image, the thickening of the small bowel was easily identified (yellow arrow in (**c**)). Only DWI images and PET maps demonstrated additional hepatic nodules, a pancreatic mass (orange arrows in (**d**,**e**) demonstrating the pancreatic lesion characterized by restricted diffusion on the axial DWI image at b 800 and the corresponding ADC map, respectively), and bone metastases (red asterisk in (**f**) on the axial DWI image demonstrating one of the bone metastases affecting the second lumbar vertebra and multiple red asterisks on the PET map in (**g**), demonstrating the high metabolic activity of the numerous skeletal lesions). Hepatic and bone marrow biopsies revealed the diagnosis of Burkitt lymphoma. The patient immediately started chemotherapy and after four cycles of rituximab, cyclophosphamide, doxorubicin, vincristine, methotrexate/ifosfamide, etoposide, and high dose cytarabine (R-CODOX-M/IVAC), a complete metabolic response occurred, as is well seen on the coronal PET map obtained after treatment (i.e., four months after the first PET/MR scan) in (**h**).

## Data Availability

Not applicable.

## References

[1] Rasper M., Kesari S. (2008). Burkitt Lymphoma Presenting as a Rapidly Evolving Cavernous Sinus Syndrome. Arch. Neur..

[2] Kalisz K., Alessandrino F., Beck R., Smith D., Kikano E., Ramaiya N.H., Tirumani S.H. (2019). An update on Burkitt lymphoma: A review of pathogenesis and multimodality imaging assessment of disease presentation, treatment response, and recurrence. Insights Imaging.

[3] Saleh K., Michot J.M., Camara-Clayette V., Vassetsky Y., Ribrag V. (2020). Burkitt and Burkitt-Like Lymphomas: A Systematic Review. Curr. Oncol. Rep..

[4] Zeng W., Lechowicz M.J., Winton E., Cho S.M., Galt J.R., Halkar R. (2009). Spectrum of FDG PET/CT findings in Burkitt lymphoma. Clin. Nucl. Med..

[5] Granero P.J., Garcia Gomez F.J., Mercado M.R., Dorado I.B. (2015). 18F-FDG PET/CT in Extranodal Burkitt Lymphoma. Clin. Nucl. Med..

[6] Giraudo C., Raderer M., Karanikas G., Weber M., Kiesewetter B., Dolak W., Simonitsch-Klupp I., Mayerhoefer M. (2016). 18F-Fluorodeoxyglucose Positron Emission Tomography/Magnetic Resonance in Lymphoma: Comparison With 18F Fluorodeoxyglucose Positron Emission Tomography/Computed Tomography and With the Addition of Magnetic Resonance Diffusion-Weighted Imaging. Investig. Radiol..

[7] Giraudo C., Karanikas G., Weber M., Raderer M., Jaeger U., Simonitsch-Klupp I., Mayerhoefer M.E. (2018). Correlation between glycolytic activity on [18F]-FDG-PET and cell density on diffusion-weighted MRI in lymphoma at staging. J. Magn. Reson. Imaging.

[8] Mayerhoefer M., Giraudo C., Senn D., Hartenbach M., Weber M., Rausch I., Kiesewetter B., Herold C.J., Hacker M., Pones M. (2016). Does Delayed-Time-Point Imaging Improve 18F-FDG-PET in Patients With MALT Lymphoma?: Observations in a Series of 13 Patients. Clin. Nucl. Med..

[9] Costa L.J., Xavier A.C., Wahlquist A.E., Hill E.G. (2013). Trends in survival of patients with Burkitt lymphoma/leukemia in the USA: An analysis of 3691 cases. Blood.

[10] Barnes J.A., Lacasce A.S., Feng Y., Toomey C.E., Neuberg D., Michaelson J.S., Hochberg E.P., Abramson J.S. (2011). Evaluation of the addition of rituximab to CODOX-M/IVAC for Burkitt’s lymphoma: A retrospective analysis. Ann. Oncol..

